# Chronic Obstructive Pulmonary Disease: Proprioception Exercises as an Addition to the Rehabilitation Process

**DOI:** 10.7759/cureus.8084

**Published:** 2020-05-13

**Authors:** Bruno Bordoni, Marta Simonelli

**Affiliations:** 1 Physical Medicine and Rehabilitation, Foundation Don Carlo Gnocchi, Milan, ITA; 2 Integrative/Complimentary Medicine, French-Italian School of Osteopathy, Pisa, ITA

**Keywords:** copd, pain, depression, diaphragm, physiotherapy, rehabilitation, fascia

## Abstract

Respiratory rehabilitation in patients with chronic obstructive pulmonary disease (COPD) is recognized as a cornerstone for the therapeutic path. Physiotherapy involves physical activity with aerobic and anaerobic exercises, which can improve the patient's symptomatic picture, such as motor function, emotional status (depression and anxiety), and improve the pain perception. The training of proprioception is not included in the structure of the exercises proposed by the Global Initiative for Chronic Obstructive Lung Disease (GOLD). The training of proprioception is a very useful strategy for stimulating the cerebellum, a neurological suffering area in patients with COPD. The cerebellum sorts information about pain and emotions, as well as motor stimuli. The article discusses the need to introduce proprioception in respiratory rehabilitation protocols, highlighting the neurological relationships with the management of comorbidities.

## Introduction and background

Chronic obstructive pulmonary disease (COPD) is the expression of multiple local and systemic disorders that culminate in chronic respiratory diseases and chronic airflow limitation; chronic cough, dyspnea, phlegm production and recurrent infections or inflammations of the bronchial tree [1,2]. Statistical projections highlight an increase in world mortality that is around 45 million deaths in the next 30 years [2]. COPD is the third cause of mortality in the world and an estimated 400 million people are affected by this chronic disease; about one in five patients, once discharged from the hospital, is still hospitalized within 30 days [3,4]. The best-known causes are the consumption of tobacco (active and/or passive), genetic predisposition, air pollution, working environment that subjects people to breathe toxic substances and/or to work in cold and humid environments [1,5]. COPD patients are subject to different comorbidities. A patient with COPD is unlikely to be free from comorbidities; in particular, if the pathology is long-lasting; metabolic, cardiovascular, neurological, psychological and psychiatric systemic diseases occur [6,7]. There is a decrease in the efficiency of neuromotor coordination, a decrease in muscle mass with phenotypic changes, a functional and morphological alteration of the diaphragm muscle and the presence of tongue disorders at night [8-10]. There is a direct relationship between muscle strength and peak inspiratory flow rate and an inverse relation between the severity of symptoms and functional capacity (physical training) [11,12]. Despite a suboptimal adherence to the physiotherapy program to improve the overall performance of the patient, we know that there is evidence on the usefulness of sending the person to carry out physical activity by health professionals (physiotherapists) [13]. Physical rehabilitation allows to improve the symptomatic picture (dyspnea and fatigue), the emotional sphere, and the quality of life (QoL) [14]. Respiratory rehabilitation through exercise is a cornerstone for the patient's path, as highlighted by the 2019 Global Initiative for Chronic Obstructive Lung Disease (GOLD) report [15,16]. The recommended physical activity is aerobic and anaerobic or mixed, depending on the study examined [17-19]. Another approach includes balance exercises, as a maintained stand on one leg or get up and sit down from a chair; the rationale is based on the fact that many COPD patients have problems of balance and fear of falling [20,21]. The causes of this inability to maintain a correct posture (daily movements) have no direct relationship with the severity of respiratory pathology (FEV1) [22]. A study has shown a causality linked to central neurological factors and the presence of a cognitive loss, probably due to cerebral vascular lesions. Impaired neuromuscular coordination, cognitive impairment may adversely affect postural control. Another element that negatively affects the functionality of the movement and the emotional status is the presence of chronic pain in these patients [23]. Pain is often localized in the chest area and in the back; pain is often not related to the presence of angina but is multifactorial [24-27]. There is a triad that worsens the patient's health status: chronic pain, depression and anxiety, and difficulty in movement [28]. The aim of the article is to highlight the need to use proprioceptive exercises in the usual rehabilitation program of COPD patients, reviewing the scientific literature about the connection between the cerebellum, emotions and perceived pain. The information we have suggests the insertion of specific exercises for the stimulation of proprioception, as it could improve the clinical picture related to pain and depression, with positive effects on muscle movement. The article could be considered to implement the physiotherapeutic approach. To conclude, the initiative could be a tool for post-COVID-19 patients in the near future; a hypothesis that still lacks data to make a statement.

## Review

Epidemiology of chronic pain, depression, and anxiety

**Figure 1:**
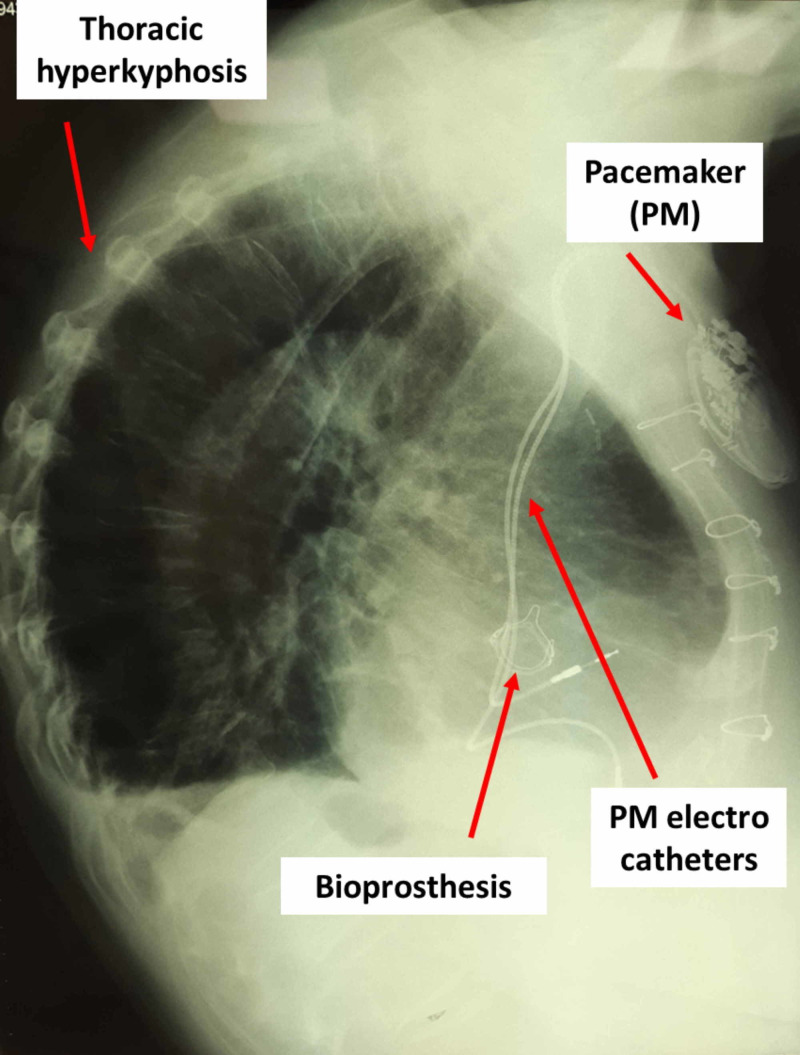
The patient with COPD shows an important dorsal hyperkyphosis, with a previous intervention in the median sternotomy for septal myectomy and aortic valve replacement with bioprosthesis, with pacemaker (PM) positioning COPD, chronic obstructive pulmonary disease

Chronic pain in COPD patients varies from 44% to 88%, with an inverse relation to the health-related quality of life (HRQoL) [24,29]. The greater the presence of pain, the lower is the physical activity performed by the patient, negatively affecting the survival rate [24]. Chronic pain is associated with depression and greater anxiety, compared to people who have no chronic pain, and with a more altered postural attitude (hyperkyphosis). Pain is one of the causes of re-hospitalization and worsening of symptoms, with increased mortality (Figure 1) [30].

An altered emotional status, such as depression and anxiety, is one of the causes for the progression of lung disease [31]. They cause re-hospitalization, worsening of symptoms, and an increase in mortality [32,33]. The percentage of presence of depression and anxiety is around 40% and 36%, respectively [32]. During the period of exacerbation of symptoms, depression rises in percentage (86%), as does anxiety (55%) [33]. Probably, the impact of the altered emotional status has a negative influence on the cognitive state.

Effects of rehabilitation on chronic pain and on emotional status

Pulmonary rehabilitation results in multiple benefits to the patient, although it can often be underappreciated compared to pharmacology [34]. The improvements may lead to changes in FEV1, functional capacity, QoL, and an improvement in the perception of pain [35]. Studies do not always directly evaluate the perception of pain, but it can be deduced from the improvement of different outcomes, as the QoL, the distance covered during the walking test; many patients suffer from chronic pathologies as osteoarthritis, presenting chronic pain [36,37]. There are several tests for the evaluation of pain, such as the Pain Sensitivity Questionnaire (PSQ), the Geriatric Pain Measure (GPM), and the Brief Pain Inventory (BPI) [38,39]. Chronic pain leads to depression and anxiety; furthermore, the fear of feeling pain leads to kinesiophobia, creating a dangerous closed circle [40]. Rehabilitation induces improvements in mood, as shown by the values of the Hospital Anxiety and Depression (HAD) test, with positive data up to 12 months post-training; the positive HAD score implements the patient's functional capacity [36]. Other data supporting the beneficial role of rehabilitation on depression and anxiety comes from the values measured by the Hamilton Rating Scale for Depression (HRSD) and the Hamilton Anxiety Scale (HAMA), the data they improve following intense activity and with patients with moderate and severe pathology [16]. The improvement of the emotional status increases the survival rates of the patient with COPD following training, even in people over 70 years of age, through the evaluation with the Beck Depression Inventory (BDI) [41].

Cerebellum, emotions, and chronic pain

Neuroimaging findings in COPD patients demonstrate structural alterations of the central nervous system [42]. The presence of chronic respiratory airway obstruction, particularly in smokers, is linked to the decrease in the cerebral gray matter; in patients with lower FEV1/FVC there is a volumetric decrease in white matter [42]. These adaptations are not necessarily linked to advancing age; moreover, these alterations reflect higher depression and anxiety values. Many studies show specific alterations, such as gray matter involved in the management of emotions (medial prefrontal cortex; anterior insula; amygdala; anterior cingulate cortex; hippocampus); other studies reveal generalized white matter lesions (myelin rarefaction, astrogliosis, axonal decrease and enlargement of perivascular spaces) [43,44]. A brain area negatively affected by the presence of COPD is the cerebellum [45]. The cerebellum sorts and manages peripheral information from the receptors related to the sensations of pain, interception, and exteroception (together with the vestibular area); this sensory information allows our brain to perceive how we are in the space in which we live, creating the motor and emotional patterns to interact. The cerebellum modulates behavioral and pain homeostasis. There is a close relationship between altered movement and behavioral and pain perception imbalances. The pain information arrives in the lobules IV-X and crus I-II (posterior area of the cerebellum), as the information arrives (from the return) also from the cortex (frontal, temporal, and parietal); the aforementioned cerebellar areas are activated even before experiencing pain [46]. The nociceptive information that reaches the cerebellum is sorted and "translated" towards the centers of the cortex and the centers of the limbic area, in a bi-univocal manner. Chronic pain reduces the size of the areas to which nociceptive information arrives [46]. The nociceptive afferent pathways travel via the spino-olivocerebellar pathway, involving the climbing fibers, the Purkinje cells; the latter also receive visceral information in the posterior cerebellar area (vermis). Pain circuits involve emotional and cognitive, motor and perceptual status [47]. Motor movement is the result of the response that the cerebellum, after it has filtered from the limbic, vestibular area, from the area of the cortex and from the entire whole/exteroceptive system, a lot of information. If, as in the case of patients with COPD, the cerebellum undergoes structural and functional alterations, there will be motor disorders connected to an incorrect perception of the nociceptive stimulus and depression [47]. As regards the cerebellum and emotions, a branch of neurological medicine names the cerebellar non-motor function that influences depression and anxiety as an "emotional cerebellum” [48]. The cerebellum processes negative and positive emotions. If there is a cerebellar lesion, negative emotions are not processed correctly (in synergy with the right hemisphere) and this will lead to aberrant emotional behaviors, such as depression [48]. The neurological circuit of depression and anxiety connects the cerebellum to different brain areas: the amygdala, hippocampus, turnip nuclei, periaqueductal gray area, pre-frontal cortical area, ventral tegmental area, and dopaminergic areas. The cerebellar areas involved in the reception of emotional and behavioral status, such as anxiety and depression, are lobules VI and VII in the neocerebellar vermis (also known as the limbic area of the cerebellum). When the patient suffers from depression and anxiety, most likely there is an alteration of the cerebellar function (hyperactivity or hypoactivity) and a reduced collaboration with the areas of the brain mentioned above. These latter brain areas have a one-to-one connection with the cerebellum. Patients entities with COPD show cerebral and structural metabolic changes, including the cerebellum; these alterations limit the information network between the different areas of the central nervous system [45].

The proprioceptivity in COPD patients

We know that there is a close relationship between movement alteration (coordination) and emotional status, as well as the processing of physical pain. We know that the COPD patient undergoes an alteration of the morphology and function of the cerebellum, as well as the important function of the cerebellar area in the management of non-motor functions is known. The cerebellum plays a primary role in managing the proprioceptive information, fundamental, among other functions, for the proper execution of the movement while maintaining balance. COPD patients show a reduction in proprioception, which condition results in less body control during daily movement with, for example, a weaker postural maintenance capacity of the ankle; the proprioceptive afferents coming from the tissues (muscles and connective tissue) are unable to be adequately processed by the cerebellum or have lost part of their role, or both conditions [30]. Inserting exercises to improve proprioception in COPD patients should be a usual procedure, through balance training, in the light of the reflections shown in the current text and for the reduced proprioceptive capacity. Studies that included balance training in the rehabilitation process (BT) showed improvements in balance and posture, indicators of improved proprioception [49,50] We do not yet have sufficient data to recommend the appropriate amount and method of administration of BT in this category of patients. Probably, the best age to administer BT is after 50 years of age, as motor imbalances are more evident from this age onwards. BT not only improves proprioceptive indices but could stimulate cerebellar areas to improve afference management body, which will be related to pain, depression, and anxiety. The pursuit of physical activity in the rehabilitation process improves motor coordination (and consequently proprioception), with improved emotional and pain threshold status [36,39,41]. Adding a specific stimulation for the cerebellar area (BT) could be a useful strategy for the implementation of the patient's well-being with COPD, to be combined with the usual rehabilitation process. Currently, there are no specific data with large numbers on the relationship of the insertion of BT in conjunction with the pulmonary rehabilitation process. Further studies are awaited.

## Conclusions

COPD patients suffer from various comorbidities, including a lower pain threshold and chronic pain, depression and anxiety. The quality of movement, understood as balance, posture and proprioceptive quality, has decreased. Studies show structural and functional alterations of the central nervous system in patients, including the cerebellum and cerebellar connections (afferents and efferences). There is a close relationship between cerebellar changes, motor changes, and the presence of non-physiological emotional status. The article has reviewed the literature concerning the possible influences of cerebellar aberrations and some comorbidities of patients with chronic lung diseases, hypothesizing to put the use of balance training in the usual rehabilitation process. The latter approach should stimulate the cerebellar areas more specifically and improve the symptomatic picture, with a wider clinical gain. Further and wider researches are needed.
